# Urban Family Planning in Sub-Saharan Africa: an Illustration of the Cross-sectoral Challenges of Urban Health

**DOI:** 10.1007/s11524-022-00649-z

**Published:** 2022-06-14

**Authors:** Trudy Harpham, Moses Tetui, Robert Smith, Ferdinand Okwaro, Adriana Biney, Judith Helzner, James Duminy, Susan Parnell, John Ganle

**Affiliations:** 1 International Union for the Scientific Study of Population, Paris, France; 2 London South Bank University, London, UK; 3 University of Waterloo, Waterloo, Canada; 4 Makerere University, Kampala, Uganda; 5 Geneva, Switzerland; 6 Aga Khan University, Nairobi, Kenya; 7 University of Ghana, Accra, Ghana; 8 University of Bristol, Bristol, UK; 9 University of Cape Town, Cape Town, South Africa; 10 J. F. Helzner Consulting, Stamford, CT USA

**Keywords:** Reproductive health, Cross-sectoral, Family planning, sub-Saharan Africa

## Abstract

The multi-sectoral nature of urban health is a particular challenge, which urban family planning in sub-Saharan Africa illustrates well. Rapid urbanisation, mainly due to natural population increase in cities rather than rural–urban migration, coincides with a large unmet urban need for contraception, especially in informal settlements. These two phenomena mean urban family planning merits more attention. To what extent are the family planning and urban development sectors working together on this? Policy document analysis and stakeholder interviews from both the family planning and urban development sectors, across eight sub-Saharan African countries, show how cross-sectoral barriers can stymie efforts but also identify some points of connection which can be built upon. Differing historical, political, and policy landscapes means that entry points to promote urban family planning have to be tailored to the context. Such entry points can include infant and child health, female education and employment, and urban poverty reduction. Successful cross-sectoral advocacy for urban family planning requires not just solid evidence, but also internal consensus and external advocacy: FP actors must consensually frame the issue per local preoccupations, and then communicate the resulting key messages in concerted and targeted fashion. More broadly, success also requires that the environment be made conducive to cross-sectoral action, for example through clear requirements in the planning processes’ guidelines, structures with focal persons across sectors, and accountability for stakeholders who must make cross-sectoral action a reality.

## Making Urban Health a Political Priority: the Illustration of Urban Family Planning

In a majority-urban world with new and recurrent health challenges, urban health should be a global political priority. The reasons for which it has fallen short of that include as follows: competition with a dominant development agenda which still has a rural focus, paucity of disaggregated urban data, lack of evidence on cost-effective interventions, and researchers’ and policy-makers’ challenges in effectively tackling the multi-sectoral nature of urban health [1]. It is this last issue on which this paper focuses. Urban family planning provides a good example of this challenge, with its necessity to join the family planning sector (as a sub-unit of the health sector) with the urban development sector, and the various routes through which the two sectors can be conceptually and empirically linked, like health, education, and employment. It will emerge that addressing the cross-sectoral impediments in this example may help ease the other above-cited hindrances to prioritizing urban health.

The source of data for this paper is a project on urban fertility and family planning in sub-Saharan Africa implemented (2018–2022) by the International Union for the Scientific Study of Population (IUSSP). The project supported mid-career African researchers to undertake research and to engage with policy-makers [2].

Towards this paper’s objective of illustrating one of the challenges of making urban health a political priority, our research question is how to begin to link family planning (FP) and urban development in sub-Saharan Africa (SSA). The ‘The Context: Fertility, FP, and Unmet Need in Urban SSA’ section describes the context of fertility, FP, and unmet need in urban SSA; the ‘The Challenge: Cross-sectoral Action Between FP and Urban Development’ section identifies challenges particular to FP and urban development in achieving cross-sectoral action; ‘The Reality: an Analysis of Cross-referencing Between FP and Urban Development in Selected SSA Countries’ section empirically measures the degrees of cross-sectoral engagement between FP and urban development in eight SSA countries by analysing cross-referencing (one sector referring to the other, in policy documents and interviews); and sections ‘An Example of Absence: Uganda’ and ‘An Example of Presence: Ghana and Kenya’ present contrasting country examples to explore the factors of strong and weak cross-sectoral policies. We use the term cross-sectoral not multi-sectoral to emphasise our focus on linking two sectors.

## The Context: Fertility, FP, and Unmet Need in Urban SSA

An additional 2.5 billion people will be added to the global urban population by 2050, of whom approximately 90% will be in Asia and SSA. The urban population of SSA is likely to almost triple. Around half of the SSA urban population lives in informal settlements [3]. Given these SSA urban population trends, it is important to consider both fertility and FP. Regarding fertility, it is now clear, though not automatically reflected in policies, that natural increase — the surplus of births over deaths amongst the residents of cities — contributes more to the growth of urban populations in low- to middle-income countries than does rural-to-urban migration [4]. In terms of FP, there is abundant evidence to show that voluntary spacing or limiting of births is important for both easing urban population growth *and* a host of well-being variables like infant, child and maternal health, female education, and female labour-force participation [5]. As with any urban health topic, it is necessary to look at both urban–rural differences and intra-urban differentials. The overall picture is that aggregate fertility in SSA is lower in urban than rural areas, *but* intra-urban differentials show the urban poor have high unmet FP need (defined as non-use of contraception amongst women wishing to limit or avoid pregnancy for at least the next 2 years). For example, studies in Kenya and Uganda indicate that women living in urban informal settlements have, perhaps surprisingly, greater unmet need than their rural counterparts [6, 7]. This comports with an important change in urban fertility trends that recent research reveals: the previous fertility-rate decline observed in many SSA cities is now stalling and even starting to reverse in some cases [8]. All this increases the imperative to understand and strengthen urban FP.

In terms of service responses to unmet need, weak governance systems affect FP supply in SSA urban areas due to fractured and confusing divisions of responsibility across the multiple levels and actors in urban governance (for a literature review of these issues, see Duminy et al.) [9].

As Harpham et al. argue, ‘We have reached a point where we cannot understand cities without understanding fertility, and we cannot understand fertility without understanding cities’, and ‘If family planning is excluded from discussions of what it means to have an urban sustainability and health agenda, then that agenda cannot be realized’ [2] (page 4).

## The Challenge: Cross-sectoral Action Between FP and Urban Development

Despite the salience of these FP facts for urban development, to what extent is the urban sector receptive to messages from the FP sector? What messages would be most effective? The IUSSP project commissioned interviews with 20 international urban experts who are mobilizing to transform urban debates and practices in different ways and at various scales, to answer these questions [10]. The results most relevant for this paper were that, first, no blanket international approach or argument will work — no ‘one size fits all’ — for at least three reasons: the fragmented nature of urban expertise and corresponding policy landscape; the unpredictable and city-specific loci of authority, energy, and drive; and the fact that the politics around both FP and urbanisation or urban governance are context-specific. ‘[N]o two cities are alike, and no two cities are going to respond to the same thing’ [10] (page 9).

The case for urban health may seem the obvious place to embed the FP argument, but it runs into the problem that the health space is perennially crowded with other issues, and recently even more so with COVID-19. Underlining FP’s manifold health benefits, for example reducing maternal mortality, can help it gain a footing in the health space. But since health itself competes for policy attention with many other concerns, urban FP advocates should capitalise on a city’s existing priorities as an entry point, i.e. connecting FP to whatever arguably-related issues already have momentum in a given city, such as climate preparedness, girls’ education, an ‘urban sustainability agenda’, or the Sustainable Development Goals (SDGs). Data is useful and necessary, but urban authorities have to be made willing to act on it.

FP in general, especially when the ‘Global North’ seems to be addressing the ‘Global South’ — ‘when the focus is exclusively placed on high fertility in the cities of the Global South’ — risks evoking some negative historical connotations with which international urban experts are wary of being associated. Also, unmet need in urban FP is not well understood: ‘Urban experts are certainly aware of government commitment to the notion of the ‘demographic dividend’ *[economic growth from an increase in the proportion of the working-age population]*… However, some may believe that factors such as education, access to basic services and cost of living, more so than access to family planning, are the principal drivers of urban fertility decline and demographic dividends’ [10] (pages 12–13).

With such context specificity of institutional and political landscape, history and attitudes around FP and urbanisation, and specific municipal concerns, global-level formulae are unlikely to offer shortcuts. Each city seems likely to need its own course of multi-stakeholder engagement, advocacy, co-production of knowledge, and co-creation of policy and programming to derive a locally successful approach to UFP.

Many of the additional factors that interviewees identified amount to political will. To summarise them, we adapt Shiffman and Smith’s framework of factors affecting political will to address global health initiatives (Table 1, columns A and B), as their categories for the more general case fit the findings for our specific case (column C) [11].

**Table 1:** Adaptation of Shiffman and Smith’s framework to urban fertility and family planning in SSA

A. Category	B. Description	C. Challenges/factors affecting political will to address urban FP issues
Actor power	Strength of the individuals involved	➢ No ‘champions’ or unifying leader (either globally or in specific cities or countries) ➢ No guiding or home institution for the topic ➢ Fragmented support, no clear academic or civil-society mobilisation
Idea-framing	How those most involved understand and portray the issue	➢ Disagreement on how to address the issue internally amongst those most involved ➢ Lack of clarity or consensus on a ‘framing’ for external audiences/decision-makers (e.g. link to ‘demographic dividend’ theory or not?)
Political contexts	Environments in which the actors operate	➢ No clear global ‘policy window’ such as a SDG or other recognised framework promoting links between fertility/family planning and urban development ➢ Many topics competing for attention within urban health; limited forums and funding to help make the case; resistance by established experts who favour their own specialties as priorities
Issue characteristics	Features of the problem or topic	➢ Limited disaggregated data ➢ Discomfort with topic of family planning (links to sex, abortion, women’s rights) ➢ Risk of neo-Malthusian views that promote population control (when linking to fertility issues) ➢ Misperceptions of the facts (stalls in urban fertility decline not recognised, misapprehensions of contributions of rural-to-urban migration versus birth rates amongst urban dwellers)

Even though the obstacles to cross-sectoral action transcend political will, the above factors are necessary context, especially insofar as there may be a two-way interaction between political will and cross-sectoral action on UFP: strong political will can overcome obstacles at more institutional levels, and in turn cross-sectoral progress on idea-framing and issue characteristics (particularly data and wider appreciation of key facts) can bolster political will.

## The Reality: an Analysis of Cross-referencing Between FP and Urban Development in Selected SSA Countries

To complement these views of international urban experts with empirical data, a search of national, current policy documents was undertaken in order to determine whether either sector was acknowledging or referring to the other at a policy level. We focused on policies rather than ad hoc urban FP programmes or projects because we were more interested in political commitment as reflected at the national policy level, whereas such discrete efforts can be initiated or stimulated by external actors (international organisations or donors), by sub-political actors such as municipal health officers, or by sub-national politicians like keen mayors. National-level political commitment indicates power to have large, possibly enduring effects. Current (2019–2021) policy documents from both the FP and urban-development sectors were examined across eight SSA countries (Burkina Faso, Ghana, Kenya, Malawi, Niger, Nigeria, Tanzania, and Uganda) by 12 IUSSP research fellows from those respective countries. In addition, a master’s student conducted a desk-based search. To assist and to complement the document search, the fellows mapped and interviewed key stakeholders from both sectors in their respective countries. Examples of such stakeholders included as follows: representatives of national-level ministries such as health and urban development; state-level officials in government (municipal assemblies) and civil society (town planning institutes); university-based academics in urban planning; and independent urban-planning professionals. In addition to asking about key policies and associated documents, the interviews asked for the respondents’ views about linking to the other respective sector. Note that all the fellows came from the FP sector and this might have introduced a bias in terms of having more knowledge and navigational skills about FP than urban development.

Only two of the countries had urban-development policies that mentioned FP (Malawi and Ghana). These were both in the context of broad development policies (which are typically formed by national planning commissions): Malawi’s Vision 2063 (2020–2063) and Ghana’s Agenda For Jobs (2018–2021) and Shared Growth and Development Agenda. The latter, for example, highlights plans to integrate family planning in all urban planning and development policies as a key national priority in national development efforts.

Why do Ghana and Malawi stand out? In the case of Ghana possibly because of:

- The availability of good-quality research on demographic issues, relative to many other countries.
- The collection of appropriate data as part of the census that allow reporting on, e.g. fertility rates relative to place of residence (urban or rural); levels, trends, and differentials in urbanisation; and drivers of migration.
- A long-running policy and political interest in population management, plus more recent efforts to mainstream population policy across other development sectors.
- A relatively strong policy and political interest in urban development, as indicated in the National Urban Policy, and interest in integrating urbanisation dynamics into the population policy.
- Significant stalls in urban fertility decline since the mid-1990s.

And in Malawi, historically one of SSA’s most densely populated countries, there has also been a successful FP programme (with strong donor interest), and more recent interest in urban development as evinced by its own national urban policy.

Five of the eight countries had FP policies that mentioned urban development (the exceptions being Uganda, Tanzania, and Burkina Faso). This asymmetry — the FP sector being more attuned to urban development than vice versa — reflects the onus of initiative and is discussed further below.

## An Example of Absence: Uganda

Of course, the mere existence of a policy, or (even more so) a reference therein, does not guarantee implementation; real cross-sectoral action may still be absent. We examined, for example, whether the extent of cross-sectoral action on urban FP in Uganda was commensurate with its Third (2020–2025) National Development Plan’s mandate for cross-sectoral action, and with statements such as that by the permanent secretary of the Ministry of Lands, Housing and Urban Development who said at a Kampala workshop on Uganda’s population challenge:

The problems associated with [urban] high fertility rates include rapid urbanisation, urban poverty, poor waste management, unemployment, environmental degradation, urban insecurity, inadequate urban infrastructure, inadequate transportation and inadequate financing. The challenges associated with high fertility rates also lead to inadequate housing, overcrowding and increased poverty and this leads to some interventions to control our population such as family planning services. It is on this basis that my ministry supports family planning interventions to reduce or control family sizes [12].

The dissonance between such a clear grasp of the issues and the fact that our research found no cross-referencing between urban planning and family planning in Uganda policy documents invites closer examination.

For this study, in-depth interviews were conducted with ten urban planning and family planning stakeholders in Uganda at various levels (four national, three urban-district, three municipal) in 2020. The interviews revealed a disconnect between the urban and family planning stakeholders. ‘There is no connection… because these are different sectors working differently. Health is doing its own work, and urban development is doing its own work’. (Family-planning interviewee, district level.) ‘No, we don’t have a direct linkage… Family planning is not of our direct interest, although we are interested in a quality population. Now there are more people coming to urban areas, and we need to be concerned about these things, otherwise our service delivery will be poor’. (Urban-planning interviewee, municipal level.) The disconnect was reinforced by what interviewees perceived as a poor, unclear means of engagement across the sectors, thereby sustaining separate ways of operating. This created a lack of appreciation of areas of common interest that could foster cross-sector collaboration. ‘The linkage with urban planners may not be very clearly spelled out. For example, what are the family-planning activities in the urban planning department? And yet urban planning is something which you can’t separate from the healthcare system where we are providing services’. (Family-planning interviewee, municipal level.) Interviewees noted that although there are existing bodies whose mandate is coordination across sectors, implementation of such coordination was lacking. ‘The national planning authority drives all of us. But sometimes you find that we are not working well across the sectors’. (Urban-planning interviewee, national level.) Coordination was described as an unimplemented mandate. Interviewees from both sectors were concerned with their core business, and generally had an inward-looking perspective. Urban-planning respondents were mainly concerned about feeding the growing population, spatial maps, making population projections, land use, and infrastructural development. ‘It’s our responsibility to make sure that we plan for the people that are projected to be here tomorrow. We get worried, but the way the government works, it’s not our mandate to control population’. (Urban-planning interviewee, national level.) ‘Urban planners don’t have any role in family planning, apart from providing spatial data based on population projections. It’s health that is concerned with family planning’. (Family-planning interviewee, district level.) Urban planners acknowledged that a slower-growing population would be preferable in order to reduce pressure on service delivery: ‘All we can do is sensitise and create awareness. We just plan, but there is pressure between population growth and general planning, and we definitely need to match our growth to the planning’. (Urban-planning interviewee, municipal level.) Neither urban nor family planning interviewees in Uganda expressed taking account of intra-urban differentials — heterogeneity amongst urban dwellers especially those living in informal settlements. Urbanisation was perceived as automatically leading to fewer children per woman; urban women were assumed to have lower fertility and a higher demand for contraceptives than their rural counterparts. Thus, urbanisation was viewed as contraception in itself.

Some interviewees in Uganda acknowledged the need for cross-sectoral collaboration. ‘There is a need for integrated planning. It would help if we talk from the same document. So, if you’re the health guy, you’re going to do health from the same document as the urban planner’. (Urban-planning interviewee, national level.) ‘Can we do it like we did for HIV prevention? Everywhere and everyone played a part…in schools, in the church, politicians, mosques etc. I think we need that kind of concerted effort by every sector’. (Family-planning interviewee, national level.) Urban planners aimed at accommodating population growth, while family planners were concerned with containing population growth. There was a lack of appreciation of the urgency for reducing urban population growth, possibly inadvertently abetted by family planning research, insofar as it has emphasised a simple rural–urban divide showing higher uptake of family planning in urban areas than rural. Although Uganda’s Third National Development Plan attributes low FP use to lack of a multi-sectoral approach to health, family planning and urban planning professionals continue to operate in silos.

## An Example of Presence: Ghana and Kenya

Stronger signs of real cross-sectoral action, or at least explicit planning therefor, are evident in these two countries, where the relationship between the FP and urban development sectors is changing. Ghana is currently revising its national urban policy, adopted in 2017 to reflect the SDGs [13]. The IUSSP project had an objective of influencing policy-makers in the urban sector and in 2021, partly as a result of research fellows’ engagement with urban policy partners, the current draft of the revised policy (which is currently undergoing review) now features a section on ‘spatially balanced and sustainable growth of urban population’. Furthermore, the urban-policy contacts whom the fellows had cultivated arranged for an FP researcher to address a review workshop and make a strong case for considering family planning and sexual and reproductive health in sustainable urban population growth and management.

An important activity proposed under this new section in the national urban policy is management of the urban population by intensifying information, education, and communication on family planning as well as on an array of sexual and reproductive health issues. A stakeholder from the Ministry of Local Government, in an interview for this study, articulated this activity’s rationale: ‘any intervention in this area [FP] that allows for improvement in the conditions of life, standard of living and providing more than just facilities [which would come under the remit of the Ministry of Health] is considered to be urban development’.

Possibilities for cross-sectoral links between FP and urban development may be present in policies and yet thwarted by complicated urban-governance politics. An example is Kenya, whose governance structure is a devolved two-tier system with the national government in charge of policy formulation and 47 county governments in charge of localisation and implementation of government policy. County-level ‘integrated development plans’ provide opportunities for cross-sectoral links between FP and urban development through the localisation of the national urban development policy and the national health policy. Nairobi county plans over two policy periods (2013–2017 and 2018–2022) address both high urban population growth and unmet need for family planning amongst the urban poor: ‘The unmet needs for family planning amongst the urban poor remains a big challenge due to the question of commodity accessibility and affordability’[14] (page 30). ‘High birth rates leads to high population therefore the county is expected to increase family planning education and services’ [15] (page 11). In 2017, a new county governor (not from the national ruling party) was elected with a concomitant desire for a new agenda and priorities. Implementation of plans by the new governor was thwarted when in 2020, the national government controversially appointed the Nairobi Metropolitan Service (NMS) to take over the running of four sectors within the county including health. The appointment of the NMS was followed by unrelenting infighting between the county government and the NMS over control of finances and functions, culminating in the national government impeaching the county governor in late 2020. Attempts to install a new governor ‘friendlier’ to the national government and NMS have stalled after the ousted governor and other interested parties obtained court injunctions. Plans and services have been severely disrupted. Thus, cross-references at the devolved policy level exist and continuous county government planning presents real possibilities for mainstreaming FP within local urban government plans; but political power struggles, reflecting the complexity of urban governance, impede implementation.

## Discussion and Conclusions

The foregoing sections have shown that there is a range of ‘uptake’ of cross-sectoral collaboration between FP and urban development. Part of the explanation for a country’s position on the range, beyond local factors, may be that the general climate in a given society or polity around discussing FP — whether it tends towards comfortable or taboo (politically, socially, culturally, religiously) — is very likely to influence discussions and eventual policy around FP expanding into urban specificities. While such climates would be difficult to measure and rank amongst countries, a proxy may be found in SDG Indicator 5.6.1, ‘Decision making on sexual and reproductive health and reproductive rights: Percentage of women aged 15–49 years who are married (or in union), who make their own decisions on three areas – their healthcare, use of contraception, and sexual intercourse with their partners’. Amongst this study’s eight countries, Kenya (56%) and Uganda (62%) rank highest on this indicator; Niger (7.3%) and Burkina Faso (20.3%) rank lowest; and the rest are clustered between 46 and 52% [16]. All of the three countries where we found that the urban development sector grasped the urban-FP issue (Ghana, Malawi, and Kenya, albeit the latter at the local not national-government level) were at or above the median amongst the eight study countries of this proxy indicator for openness of FP discourse. Uganda, ranking highest in this indicator, as described above in the ‘An Example of Absence: Uganda’ section evinces a solid understanding of the issue at the political level though it falters at implementing cross-sectoral action. This apparent correlation between cultural attitudes and progress of UFP is a context to be borne in mind.

The asymmetry of FP cross-referencing urban more than vice versa suggests that awareness of the need for cross-sectoral collaboration on UFP is greater in the FP sector. Current bodies of evidence should suffice to sway both sides and move each towards the other — that FP cannot succeed unless it succeeds in cities, and that urban development will struggle unless it addresses urban population growth and unmet need for FP. But it is the FP side that is producing the evidence, and thus perforce is more aware of it. Also, FP is more bounded, discrete, and professionally coherent than the sprawling urban-governance domain which must encompass not only many sectors but also the dimensions of politics, culture, technocracy, and bureaucracy. In trying to penetrate urban governance to raise its awareness, FP contends with several other specialisations that also have evidence that their sector can contribute to urban development, and are similarly vying for attention in the complex urban domain.

**Fig. 1:**
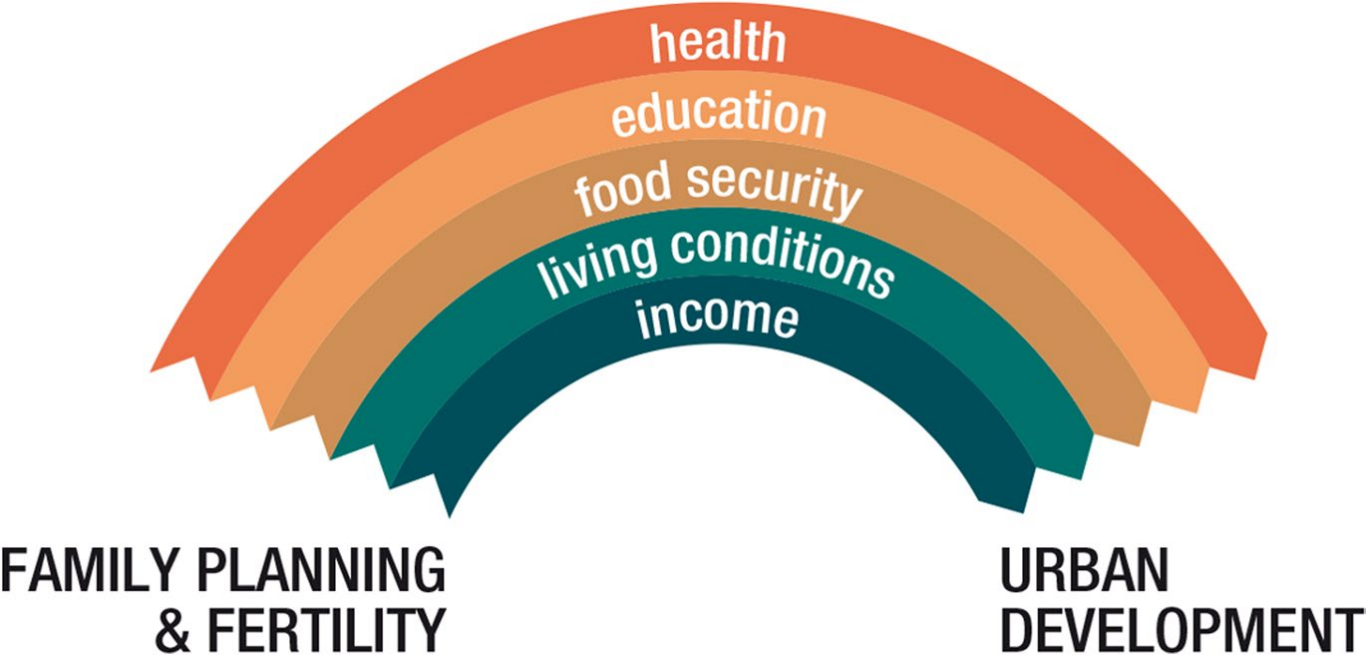
Family planning and fertility’s pathways of benefit to urban development

Nonetheless, this same urban complexity offers multiple arguments and potential entry points for advocates of urban FP, armed with evidence that FP contributes positively to several urban-governance preoccupations (Fig. 1) [17]. The message from the FP sector can be packaged in various ways, e.g. using health, education, or environment-related evidence and arguments; and indeed in this study’s reviewed documents and interviews, the urban sector mentioned several of these arcs. The FP sector needs to capitalise on this more — capturing each city’s concerns, framing the message and associated path of arguments and evidence according to country and city context and policy priorities. Also, although the arrows in this figure go from left to right (FP to urban), there may also be two-way causal flow, i.e. urban improvements in these areas may in turn facilitate FP.

Furthermore, to defuse arguments that greater resources to UFP would subtract from other urban priorities, UFP advocates may wish to emphasise FP’s potential cost-effectiveness: according to a study of low- and middle-income countries (though not differentiating between urban and rural), ‘Every dollar spent on contraceptive services beyond the current level would reduce the cost of pregnancy-related and newborn care by three dollars’ [18] (page 5). Such an argument could infer savings beyond the health sector, in that slower population growth would ease a myriad of urban challenges — those listed in Fig. 1, and many others such as poverty, waste management, unemployment, environmental degradation, insecurity, and inadequacies of infrastructure and transportation.

Finding the right pathways for advocacy to emphasise is a cross-sectoral undertaking: FP specialists need the insights and commitment of urban-development specialists (technical and political) to formulate the arguments most likely to succeed in a given urban polity. This study’s finding that a city’s preoccupations (and potential lines of argument for UFP advocacy) tend to be distinct, and therefore advocacy strategy and entry points will differ from city to city, does not imply that the importance of cross-sectoral collaboration is uneven. Solid evidence on and coherent advocacy for UFP are necessary in any context, and cross-sectoral collaboration may be a necessary if insufficient condition for both.

As a practical matter, successful cross-sectoral advocacy for urban family planning requires not just solid evidence, but also internal consensus and external advocacy. FP actors and their urban-development collaborators must cohere to agree on the best framing of the issue per local preoccupations, and then communicate the resulting key messages in targeted and concerted fashion.

More broadly, success also requires that the environment be made conducive to cross-sectoral action, for example through clear requirements in the planning processes’ guidelines, structures with focal persons across sectors, and accountability for stakeholders who must make cross-sectoral action a reality.

In the ‘Making Urban Health a Political Priority: the Illustration of Urban Family Planning’ section, we suggested that addressing the cross-sectoral impediments in this UFP example may help ease other hindrances to prioritizing urban health — a predominantly rural development paradigm, paucity of disaggregated urban data, and lack of evidence on cost-effective interventions. A cross-sectoral espousal of UFP, against prevailing currents that focus FP on rural populations, is a step towards balancing urban and rural development paradigms. Cross-sectoral collaboration on UFP can inform design of the collection and analysis of disaggregated urban data. Spreading UFP practice will offer more opportunities to assess interventions’ cost-effectiveness. Cross-sectoral collaboration on UFP will not be a keystone that solves all the other challenges of urban health, within and beyond UFP; but unblocking such collaboration is likely to have complementary effects on the other challenges.

This study’s chief limitations are a relatively opportunistic sample of countries (being where project fellows are based), and the fact that UFP is unevenly developed in them. The latter has at least two implications for this and future studies: first, a paucity of successes, and second, a dearth of organised information that would allow more objective cross-country analysis. Nonetheless, this study has found several elements that address its research question of how to begin to link FP and urban development in SSA: the need to understand the challenges (which inhere variously in FP, in urban development, and/or in cross-sectoral collaboration generally or specifically between these two sectors); institutional and policy measures to overcome cross-sectoral barriers; and how to capitalise on urban complexity by identifying the evidence and arguments most likely to resonate beyond the health sector. These findings on strategies to win attention and multi-sectoral action amidst crowded urban-governance agendas can be relevant to urban health in general, and in particular to urban health problems such as urban mental health and gender-based violence. These, like urban FP, are culturally sensitive, need a degree of behavioural and/or social change, and (with their multiple determinants and intervention paths) require multi-sectoral action.
